# Human iPSC-derived retinal organoids develop robust Alzheimer’s disease neuropathology

**DOI:** 10.3389/fncel.2024.1340448

**Published:** 2024-01-23

**Authors:** Ethan James, Anne Vielle, Karen Cusato, Helen Li, Byoungin Lee, Shama Parween, Anna Howell, Noah R. Johnson, Heidi J. Chial, Huntington Potter, M. Natalia Vergara

**Affiliations:** ^1^CellSight Ocular Stem Cell and Regeneration Research Program, Sue Anschutz-Rodgers Eye Center, University of Colorado School of Medicine, Aurora, CO, United States; ^2^Linda Crnic Institute for Down Syndrome, University of Colorado Anschutz Medical Campus, Aurora, CO, United States; ^3^University of Colorado Alzheimer’s and Cognition Center, University of Colorado Anschutz Medical Campus, Aurora, CO, United States; ^4^Department of Neurology, University of Colorado Anschutz Medical Campus, Aurora, CO, United States

**Keywords:** hiPSC, retina, organoid, Alzheimer’s disease, disease modeling

## Abstract

Alzheimer’s disease (AD), characterized by memory loss and cognitive decline, affects nearly 50 million people worldwide. Amyloid beta (Aβ) plaques and intracellular neurofibrillary tangles (NFTs) of phosphorylated Tau protein (pTau) are key histopathological features of the disease in the brain, and recent advances have also identified AD histopathology in the retina. Thus, the retina represents a central nervous system (CNS) tissue highly amenable to non-invasive diagnostic imaging that shows promise as a biomarker for early AD. Given the devastating effects of AD on patients, their families, and society, new treatment modalities that can significantly alter the disease course are urgently needed. In this study, we have developed and characterized a novel human retinal organoid (RO) model derived from induced pluripotent stem cells (iPSCs) from patients with familial AD due to mutations in the amyloid precursor protein gene (APP). Using immunofluorescence and histological staining, we evaluated the cellular composition and AD histopathological features of AD-ROs compared to control ROs from healthy individuals. We found that AD-ROs largely resemble their healthy control counterparts in cellular composition but display increased levels of Aβ and pTau. We also present proof of principle of an assay to quantify amyloid levels in whole ROs. This *in vitro* model of the human AD retina constitutes a new tool for drug screening, biomarker discovery, and pathophysiological studies.

## 1 Introduction

Worldwide, 15% of the population above the age of 65 years, the equivalent of nearly 50 million people, suffers from Alzheimer’s disease (AD) (Knopman et al., 2021), and cases are projected to increase to 113 million worldwide by 2050 (Wu et al., 2017). AD is a neurodegenerative disorder most prominently characterized by dementia and sensory system dysfunction, and it is histopathologically defined by the presence of amyloid beta (Aβ) plaques as well as intraneuronal neurofibrillary tangles (NFTs) of hyperphosphorylated Tau protein (pTau) (Cras et al., 1991; Price et al., 1991).

Pathological Aβ peptides are produced by processing of the amyloid precursor protein (APP) through cleavage by β- and γ-secretases (De Strooper and Scorrano, 2012). In some familial forms of AD, APP duplication, mutations in APP, or mutations in components of γ-secretase such as Presenilin 1 (PSEN1) or Presenilin 2 (PSEN2) result in increased production of Aβ (Scheuner et al., 1996; Murayama et al., 1999). The “amyloid cascade hypothesis” states that the oligomerization of Aβ into neurotoxic species and its aggregation into plaques initiates a chain of events resulting in neuroinflammation, neurodegeneration, memory loss, and senile dementia by impairing synaptic function and leading eventually to cell death (Hardy and Higgins, 1992; Shankar et al., 2007, 2008; Walsh et al., 2007; Knopman et al., 2021). Additionally, Tau, a microtubule-associated protein present in axons and pre- and post-synaptic compartments, is hyperphosphorylated in AD, and tends to aggregate, forming toxic species that can propagate through synapses, impair axonal transport, and lead to the formation of NFTs (Wu et al., 2016; Gallardo and Holtzman, 2019; Knopman et al., 2021).

Notably, AD pathology is found not only in the brain but also in the retinas of AD patients. This includes the presence of Aβ deposits and plaques, pTau, NFTs, and signs of neuroinflammation in post-mortem AD retinas (Koronyo et al., 2023). Since the retina is an easily accessible part of the central nervous system (CNS) and there is a wide array of non-invasive ophthalmic imaging technologies, retinal features of AD are being evaluated as potential biomarkers for disease diagnosis and for the evaluation of disease progression (Hadoux et al., 2019; Koronyo et al., 2023).

There is a critical need to develop effective therapies for AD that can improve the quality of life for patients and ameliorate the individual and societal burden of the disease. Unfortunately to date, there are no therapies that can stop or reverse the progression of AD (Cummings et al., 2014; Kusne et al., 2017). This paucity is due, at least in part, to the limitations of animal models used for drug development in effectively recapitulating human AD pathology.

In this context, retinal organoid (RO) models derived from human induced pluripotent stem cells (hiPSCs) may bridge this gap by complementing the current AD drug development pipeline. ROs mimic, to a large extent, the native retinal tissue structure and cellular composition and present some advantages over the use of brain organoids for the development of drug screening assays, including: higher reproducibility, tissue transparency, and the availability of fast and quantitative screening technologies applicable to whole organoids that preserve tissue architecture and can enable longitudinal studies (Aasen and Vergara, 2020). Moreover, some features of AD pathology have recently been described in 5-month-old hiPSC-ROs derived from patients with mutations in the PSEN1 and PSEN2 genes, which cause early onset familial AD (EOFAD) (Lavekar et al., 2023). Namely, increased pTau was noted in RO tissue and protein extracts, and an increase in the Ab42:Ab40 ratio was found in conditioned medium from these ROs.

To expand upon these findings and develop alternative human RO models of AD to better approximate the range of EOFAD mutations, we used two patient-derived hiPSC lines, one line with an APP duplication that results in cells with three copies of the APP gene, and one line harboring the “London” mutation in the APP gene, and we combined them with a well-established protocol for the generation of ROs (Zhong et al., 2014). To validate this model, we evaluated the cellular composition of AD-ROs compared to control ROs derived from sex-matched healthy donors and quantified the presence of amyloid, pathological Aβ aggregates, and pTau in the retinal tissue using histological techniques. We show evidence that AD-ROs resemble control ROs in their architecture and cellular composition and that they recapitulate the main histopathological hallmarks of AD as early as 3 months of differentiation. We also provide proof of concept of the possibility to quantify amyloid in whole ROs. We expect that this model will constitute a valuable new screening tool in the translational research arsenal to develop early AD biomarkers and test potential therapeutic agents.

## 2 Materials and methods

### 2.1 Cell lines and retinal organoid generation

Two patient-derived AD lines were used to generate ROs. The female fibroblast-derived UCSD239i-APP2-1 cell line (WiCell, WB66585) carries a duplication of the APP gene (Israel et al., 2012). The male, fibroblast-derived HVRDi001-A-1 cell line (WiCell, WB66254) is heterozygous for the p.Val717Ile (c.2149G>A) “London” mutation in the APP gene (Muratore et al., 2014). Two healthy control lines were used: the female, cord blood-derived episomal hiPSC line A18945 (Gibco), and the male, urine-derived, RNA-reprogrammed hiPSC line iLC42-3 (University of Colorado). All hiPSC lines were seeded on growth factor reduced Matrigel basement membrane matrix (Corning, 354230) and maintained in mTeSR1 medium (StemCell Technologies). Cells were passaged at 60%–70% confluence.

To generate ROs, we followed a previously published protocol (Zhong et al., 2014). Briefly, hiPSC colonies were detached with dispase and grown in suspension to form embryoid bodies (EBs). Cultures were then gradually transitioned from mTeSR1 medium containing 10 μM Rock inhibitor (Blebbistatin, Selleckchem) to neural induction medium containing DMEM/F12 (1:1), 1% N2 supplement (Invitrogen), 1× minimum essential media-non-essential amino acids (NEAAs), 2 μg/ml heparin (Sigma) over 3 days. At D7, EBs were plated on Matrigel, and on D16, the medium was changed to retinal differentiation medium [DMEM/F12 (3:1)] supplemented with 2% B27 (without vitamin A, Invitrogen), 1× NEAA and 1% antibiotic–antimycotic (Gibco). Retinal domains were identified under phase-contrast microscopy and manually detached between D21 and D25 for suspension culture. At D30, the medium was switched to DMEM/F12 (3:1) supplemented with 2% B27 (without vitamin A, Invitrogen), 1× NEAA, and 1% antibiotic–antimycotic, 10% fetal bovine serum (FBS; Gibco), 100 mM Taurine (Sigma), and 2 mM GlutaMAX (Invitrogen). Starting on D63, 1 mM retinoic acid was added daily. On D91, the medium was changed to DMEM/F12 (1:1) supplemented with 1% N2, 1× NEAAs, 1% antibiotic–antimycotic, 10% FBS, 100 mM Taurine and 2 mM GlutaMAX, and retinoic acid supplementation was decreased to 0.5 μM. The use of hiPSCs in this study conforms to the University of Colorado Institutional Biosafety Committee standards.

### 2.2 Immunofluorescence and amyloid staining on tissue sections

Retinal organoids were collected at 3 and 5 months of differentiation, washed three times in PBS, and fixed in 4% paraformaldehyde (PFA) for 10 min at room temperature, followed by three washes in 1× PBS. Samples were then subjected to a sucrose gradient (6.12%, 12.5%, and 25%) before embedding in a 1:1 OCT:25% sucrose solution for cryoprotection. A total of 10 μm sections were obtained using a Leica cryostat and processed for immunofluorescence or stored at −20°C until used. For indirect immunofluorescence staining, slides were washed three times in PBS and blocked in 10% donkey serum (DS) in 0.25% PBST for 1 h at room temperature. After blocking, primary antibodies were diluted in 2% DS in 0.25% PBST and incubated overnight at 4°C (Table 1). Slides were then washed three times in 0.25% PBST and incubated with secondary antibody diluted at 1:3,000 in 2% DS, 0.25% PBST for 1 h at room temperature (Table 1). Slides were then washed three times for 5 min in 0.25% PBST followed by one 5-min wash in 1X PBS, all at room temperature. Nuclei were counterstained with DAPI before mounting the slides using Fluoromount-G (Fisher Scientific). For NIAD-4 staining, immunostained slides were incubated with NIAD-4 (Cayman Chemical, 18520) diluted 1:1,000 in PBS for 10 min at room temperature, washed three times in 1X PBS, counterstained with DAPI, and mounted with Fluoromount-G.

**TABLE 1:** Antibodies used in this study.

Antibodies used in immunofluorescence experiments				
**Antigen**	**Manufacturer**	**Catalog number**	**Source**	**Dilution**
Otx2	Millipore	AB9566I	Rabbit	1/200
Recoverin	Millipore	AB5585	Rabbit	1/1,000
PROX1	DSHB	1A6	Mouse	1/60
AP-2-alpha	DSHB	3B5	Mouse	1/40
ISL1/2	DSHB	39.4D5	Mouse	1/50
HuC/D	Invitrogen	A21271	Mouse	1/100
RBPMS	Proteintech	15187-1-AP	Rabbit	1/500
RXR-gamma	Santa Cruz Biotechnology	sc-365252	Rabbit	1/500
NRL	R&D Systems	AF2945	Goat	1/300
PKC alpha	Proteintech	21991-1-AP	Rabbit	1/500
RLBP1	Proteintech	15356-1-AP	Rabbit	1/800
Aβ17-24 (4G8)	BioLegend	800712	Mouse	1/200
pTau (AT8)	Thermo Fisher Scientific	MN1020	Mouse	1/150
pTau (T231)	Abcam	ab151559	Rabbit	1/200
Mouse IgG-Fc	Invitrogen	A10036	Donkey	1/3,000
Rabbit IgG-Fc	Invitrogen	A21206	Donkey	1/3,000
Goat IgG-Fc	Invitrogen	A11055	Donkey	1/3,000

### 2.3 Image analysis and quantification

Fluorescence images were acquired with a Nikon C2 Confocal Microscope A1 (Minato City, Tokyo, Japan) using the same parameters for all images, and were stitched together using Nikon Elements Software. Images were analyzed with Fiji (Bethesda, MD, USA). A minimum of five organoids per hiPSC line were analyzed for each marker. The relative area of positive immunostaining was calculated as a percentage of total retinal area (labeled area/DAPI-stained area). This allowed for the quantification of the proportion of different cell types or immunolabeling within the retina, thus accounting for potential differences in organoid size. For colocalization analysis of 4G8 and NIAD-4, the area of double labeling was measured with Fiji and expressed as a percentage of total NIAD-4 (+) area. Analyses were performed blinded to RO control or AD status.

### 2.4 Quantitative 3D amyloid staining assay

Retinal organoids at 3 months of differentiation (*n* = 6 per condition), were fixed for 1 h in 4% paraformaldehyde and washed three times in PBS. Organoids were then plated in a 96-well, black V-bottom plate at one organoid per well in 1X PBS, and the auto-fluorescent background was measured by 3D-automated reporter quantification (Vergara et al., 2017; Vielle et al., 2023). Briefly, a Tecan Spark Plate reader (application SparkControl v2.3) was used, and after determining the Z-positions, fluorescence intensity was measured using the following optimized parameters: Mode: Fluorescence Top Reading; Excitation wavelength: 475 nm; Excitation bandwidth: 5 nm; Emission wavelength: 625 nm; Emission bandwidth: 5 nm; Gain: 100, Manual; Number of flashes: 20; Integration time: 40 μs; Lag time: 0 μs (Fluorobrite DMEM filled wells were used as blank). Organoids were then stained for 1 h with 10 μM NIAD-4 (Cayman Chemical) at room temperature. The plate fluorescence intensity was reassessed using the same parameters. For size normalization (Vergara et al., 2017; Vielle et al., 2023), nuclear staining with 10 μg/ml Hoechst 33342 (Invitrogen, H3570) was performed for 1 h at room temperature, followed by three 15 min washes in 1X PBS. After three 15 min washes, the organoids were incubated overnight in 80% glycerol at 4°C, washed 3 × 15 min in PBS, washed a final time in DMEM Fluorobrite, and fluorescence intensity was assessed using excitation wavelength: 350 nm; Excitation bandwidth: 5 nm; Emission wavelength: 461 nm; Emission bandwidth: 5 nm. Confocal images of whole ROs were taken as described above. Fluorescence intensity was measured post-clearing, background was subtracted, and NIAD-4 intensity was normalized to Hoechst staining to correct for size differences (Vergara et al., 2017).

### 2.5 Statistical analyses

Statistical analyses were performed with GraphPad Prism 10.1.0 Software using Mann–Whitney test for pair-wise comparisons (no assumption of normal distribution). A *p*-value < 0.05 was considered statistically significant. Bar graphs depict the mean ± standard error of the mean (SEM), and individual values are plotted. Outliers were identified by Rout method and removed prior to Mann–Whitney test.

## 3 Results

### 3.1 Familial AD hiPSCs can generate retinal organoids with all major retinal cell types

With the goal of generating new human RO models of histopathology from EOFAD patients with APP mutations, we used two different hiPSC lines derived from patients with familial forms of AD: UCSD239iAPP2-1 (F, APP duplication) and HVRDi001-A-1 (M, p.Val717Ile “London” mutation in APP). We used two healthy control hiPSC lines (A18945 and iLC42-3) in parallel to validate our findings. We first assessed the ability of the two AD lines to generate retinas *in vitro* using a well-established and validated protocol for RO generation (Zhong et al., 2014), and found that all of the control and AD hiPSC lines showed the capacity to generate ROs. Even though not systematically quantified, no major differences were noted in the ability of the cell lines used to generate ROs (efficiency of organoid generation). Thus, we proceeded to characterize the model by evaluating whether it develops the full range of retinal cell types.

**FIGURE 1:**
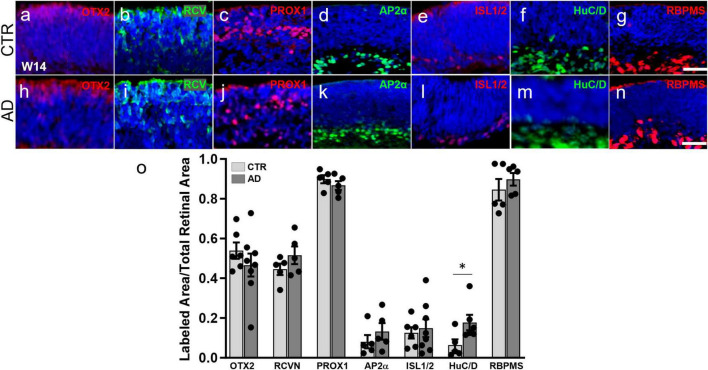
Alzheimer’s disease hiPSC lines produce retinal organoids with major retinal cell types and in a pattern similar to control organoids. **(a–n)** Immunofluorescence for markers of different retinal cell types at 3 months of differentiation in cryosections of AD and control (CTR) organoids shows that all early born retinal cell types are generated. Markers used were: OTX2 **(a,h)** and recoverin (RCVN) **(b,i)**, for photoreceptor precursors; PROX1 **(c,j)** for horizontal and amacrine cells; AP2α **(d,k)** for amacrine cells; ISL1/2 **(e,l)** for amacrine and retinal ganglion cells; HuC/D **(f,m)**, for amacrine and ganglion cells; and RBPMS **(g,n)** for retinal ganglion cells. **(o)** Immunofluorescence quantification shows no significant differences in OTX2, recoverin, PROX1, AP2α, ISL1/2, and RBPMS. Mann–Whitney test; *p < 0.05; *N* ≥ 5 organoids per condition including samples from UCSD239iAPP2-1 and HVRDi001-A-1 (AD lines), and A18945 and ILC42.3 (control lines). Scale bars, 50 μm. Bars represent mean ± SEM (error bars).

Since ROs follow a characteristic developmental pattern that resembles that of the *in vivo* retina (Vielle et al., 2021), we collected organoids at 3 months of differentiation, a stage when all early-born cell types have already undergone their peak of differentiation in humans as well as in human ROs. Immunohistochemical analysis on organoid sections for OTX2 and recoverin (photoreceptor precursor markers at this time point), showed a similar pattern and cell numbers in control (CTR) and AD ROs (Figures 1a, b, h, i, o). The same was true of markers of interneurons (PROX1, horizontal, and amacrine cells; AP2α, labeling a range of amacrine cell subpopulations) (Figures 1c, d, j, k, o). Additionally, no significant differences were observed in ISL1/2, a marker of cholinergic amacrine cells and ganglion cell subpopulations at this developmental stage (Figures 1e, l, o), and in RBPMS, a marker of ganglion cells (Figures 1g, n, o). However, we did observe a small but significant increase in HuC/D labeling (ganglion and amacrine cells), from 0.064% ± 0.029% of retinal area in CTR-ROs to 0.177% ± 0.038% in AD-ROs (mean ± SEM; Figures 1f, m, o). Further studies are needed to elucidate the nature of the cells and the mechanisms responsible for this increase.

We then analyzed whether AD-ROs produce late-born retinal cells (Supplementary Figure 1). For this purpose, we continued to culture ROs derived from A18945 (CTR) and HVRDi001-A-1 (AD) lines until 5 months of differentiation and performed immunofluorescence staining. We identified the presence of PKCα (rod bipolar cells, Supplementary Figure 1c, g) and CRALBP (Müller glia, Supplementary Figure 1d, h) in a similar pattern in AD and CTR ROs. We also quantified NRL (+) cells (rod photoreceptors) and RxRγ (+) cells in the outer nuclear layer area (early cone photoreceptors) and found no significant difference among conditions (Supplementary Figure 1a, b, e, f, i), suggesting that AD genotype does not alter photoreceptor subtype ratios in the AD-RO model.

Together, our results indicate that hiPSC-derived RO models of familial AD recapitulate, to a great extent, the structure and cellular composition of organoids from healthy control hiPSC lines and of the native human retina.

### 3.2 AD retinal organoids exhibit amyloid and Tau pathology

**FIGURE 2:**
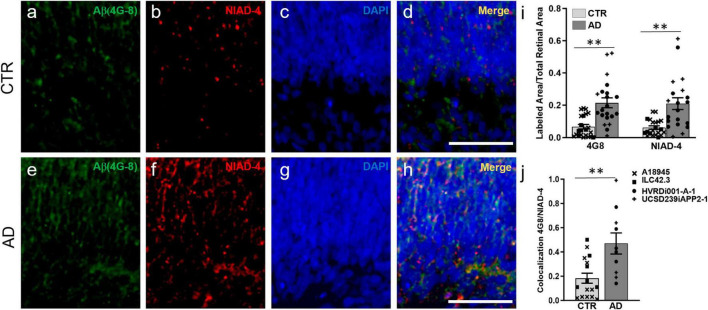
Alzheimer’s disease retinal organoids exhibit increased Aβ deposits. Immunofluorescence and histological staining at week 14 of differentiation in cryosections of CTR-ROs **(a–d)** and AD-ROs **(e–h)**. AD-ROs label for Aβ (4G8; **a,e**) and for NIAD-4, a histological stain for amyloid **(b,f)**, as noted by the increased labeling in panels **(e,f)**. All samples were counter-stained with DAPI **(c,g)** and increased colocalization of 4G8 and NIAD4 was noted in AD-ROs **(d,h)**. **(i)** Image quantification shows statistically significant differences between AD-ROs and CTR-ROs in 4G8 immunolabeling and NIAD-4 staining. **(j)** Colocalization of 4G8 and NIAD staining as a proportion of total NIAD-4 (+) area in AD-ROs and CTR-ROs. *n* = minimum 10 per condition, including samples from UCSD239iAPP2-1 and HVRDi001-A-1 (AD lines), and A18945 and ILC42.3 (control lines); Mann–Whitney test, **P < 0.01. Scale bars, 50 μm. Bars represent mean ± SEM (error bars). Individual data points are as indicated in graph legend.

We investigated whether AD-ROs could recapitulate some of the major hallmarks of AD histopathology observed in the human retina and brain. Specifically, we investigated the presence and accumulation of Aβ peptides and hyperphosphorylated forms of Tau, some of the key histopathological features of the disease. We chose 3 months of differentiation for our analyses, as retinal ganglion cells are lost from organoid models after this time (Cowan et al., 2020; Vielle et al., 2021), and because amyloid has been found at this time point in other human stem cell-derived *in vitro* models of the CNS (Raja et al., 2016; Zhao et al., 2020). Immunofluorescence staining and image analysis of organoid sections with a commonly used and well validated antibody for Aβ, 4G8, showed a significant increase in Aβ deposition in AD-ROs compared to CTR-ROs (Figures 2a, c–e, g–i). Additionally, staining for NIAD-4, a fluorescent amyloid binding dye that works by intercalating into beta-pleated sheet structures, also showed a significant increase in amyloid deposition in the AD-ROs compared to CTR-ROs, suggestive of amyloid formation (Figures 2b–d, f–i). Additionally, NIAD-4 staining co-localized with Aβ (4G8) staining at significantly higher rates in AD-ROs than in CTR-ROs, indicating the presence of dense-core amyloid plaques (Figure 2j).

**FIGURE 3:**
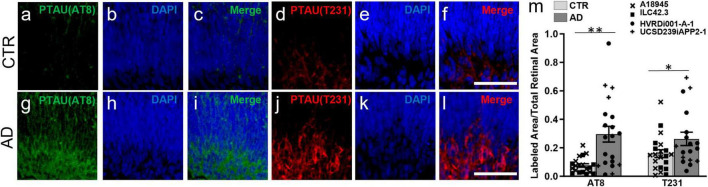
Alzheimer’s disease retinal organoids exhibit increased pTau labeling. **(a–l)** Immunofluorescence staining using antibodies against pTau, AT8 **(a,c,g,j)** and T231 **(d,f,i,j)**. All cryosections were counterstained with DAPI **(b,e,h,k)**. **(m)** Quantification of AT8 and T231 immunolabeling shows increased pTau in AD-ROs compared to CTR-ROs. *n* = minimum 10 per condition, including samples from UCSD239iAPP2-1 and HVRDi001-A-1 (AD lines), and A18945 and ILC42.3 (control lines); Mann–Whitney test, *p < 0.05, **P < 0.01. Scale bars, 50 μm. Bars represent mean ± SEM (error bars). Individual data points are as indicated in graph legend.

Moreover, we also found increased Tau phosphorylation in AD-ROs compared to CTR-ROs using antibodies against two different phosphorylated forms of Tau: AT8 (phosphorylation in Ser202, Thr205; Figures 3a–c, g–i), and T231 (phosphorylation in Thr231; Figures 3d–f, j–l). Immunofluorescence quantification showed an increase in both markers in AD-ROs (Figure 3m).

These results support the validity of this new RO model to investigate increased Aβ and pTau, two hallmarks of AD pathology.

### 3.4 Fluorescence-based assay for the quantitative assessment of amyloid plaques in retinal organoids

**FIGURE 4:**
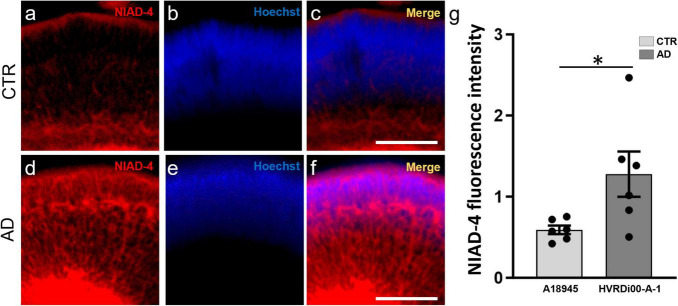
Quantitative assay to assess amyloid levels in 3D retinal organoids. Whole-mount retinal organoids (3 months post-differentiation) were stained with NIAD-4 for amyloid and with Hoechst, which labels all cells, for size normalization. **(a–f)** Whole mount imaging of stained organoids exemplifies the pattern of expression. Note the increased labeling in the AD-ROs **(a,c)** compared to CTR-ROs **(d,f)**. **(g)** Total NIAD-4 fluorescence intensity was quantified in whole ROs using 3D-automated reporter quantification on a Tecan Spark multi-well plate reader and normalized to Hoechst after background subtraction. Bar graph shows significant increases in NIAD-4 labeling in AD-ROs (HVRDi001-A-1) compared to CTR-ROs (A18945) (individual data points represented by circles). *n* = 6 per condition; Mann–Whitney test, *p < 0.05. Bars represent mean ± SEM (error bars).

Drug development and biomarker validation applications necessitate both a robust model and simple, sensitive, and quantitative assays for outcome measures. To provide proof of principle of the ability to perform quantitative analyses of amyloid in whole 3D ROs, we made use of a technology we previously developed and validated termed 3D Automated Reported Quantification (3D-ARQ) (Vergara et al., 2017; Vielle et al., 2023). This technology is based on the quantification of total fluorescence intensity with high speed and high sensitivity. We had also identified RO size as the major source of variability in fluorescence intensity outputs in ROs and devised a method for size normalization using a globally expressed fluorophore (Vergara et al., 2017; Vielle et al., 2023). Therefore, we performed whole mount staining of CTR-ROs (A18945) and AD-ROs (derived from the HVRDi001-A-1 line) after 3 months of differentiation with NIAD-4 to stain amyloid, and we determined the best parameters for 3D-ARQ quantification of total fluorescence intensity using a TECAN Spark plate reader as described in the Methods. Hoechst 33342 staining was used for size normalization and background fluorescence of the media was subtracted. The results of these experiments showed increased NIAD-4 fluorescence in AD-ROs over CTR-ROs (Figures 4a–f) and these differences were statistically significant (Figure 4g).

## 4 Discussion

Alzheimer’s disease leads to significant morbidity and mortality worldwide. While AD is notable for its associated cognitive decline and the presence of Aβ plaques, pTau, and NFTs in the brain, AD pathology is also present in the post-mortem human retina in a manner that appears to correlate with brain pathology and cognitive measures (Koronyo et al., 2023). While post-mortem human CNS tissues are useful for histological and biochemical studies, their utility in mechanistic studies, longitudinal studies, and drug discovery are limited. Here, we have established a novel human AD model using ROs grown from iPSCs from patients with EOFAD due to APP mutations. These AD-ROs recapitulate normal retinal development with immunolabeling showing similar complements of retinal cell types and numbers. The only significant difference noted was in HuC/D labeling, which is found in retinal ganglion cells, amacrine cells, and early horizontal cells (Ekstrom and Johansson, 2003). ISL1/2, which labels subpopulations of ganglion cells and a subset of amacrine cells (Stanke et al., 2008; Martin-Partido and Francisco-Morcillo, 2015; Luo et al., 2019), was not significantly different between CTR-ROs and AD-ROs, and neither were RBPMS, which is expressed in ganglion cells (Rodriguez et al., 2014), and AP2α, which is reported to label a range of amacrine cell subpopulations and early developing horizontal cells (Bassett et al., 2007). It is possible that the differences observed in HuC/D may represent specific subsets of ganglion and/or amacrine cells that were increased in number. Alternatively, there may be slight differences in ganglion and/or amacrine cell markers that our sample size was too small to detect.

Importantly, we found that our AD-RO model also recapitulates AD pathology with increases in Aβ and pTau. While we were putting the finishing touches on this manuscript, a study was published that described the generation of AD-ROs from patients with PSEN mutations (Lavekar et al., 2023). The authors showed an increase in AT8 labeling of pTau in the inner retina of AD-ROs at 5 months of differentiation, as well as an increase in the secreted Ab42:40 ratio by ELISA in conditioned media from AD-ROs compared to ROs derived from control hiPSC lines. Moreover, they mentioned that subtle changes in those phenotypes were also noted in the PSEN-AD cell lines as early as 3 months (Lavekar et al., 2023). Our results confirm and expand upon these findings using a new model of the AD retina derived from hiPSC with APP mutations and showing robust retinal AD histopathology at 3 months of differentiation. We found increases in Tau phosphorylation in Ser202, Thr205 and in Thr231 by immunofluorescence staining (AT8 and T231, respectively), as well as deposition of Aβ in retinal tissues by immunofluorescence for 4G8 and staining for NIAD-4 in AD-ROs compared to CTR-ROs. Colocalization of 4G8 with NIAD-4 indicates that amyloid protein is indeed deposited into plaques in AD-ROs with APP mutations. This human retinal model of AD will be useful for developing new AD biomarkers, studying disease mechanisms, and testing potential therapeutics. However, it should be noted that one of the limitations of this and previous studies is that, even though multiple AD and control hiPSC lines have been used, the control lines have been derived from unrelated individuals and from different cellular sources, and that the development and use of isogenic pairs in future studies would be an important additional validation.

Currently, diagnosis of AD requires loss of memory noted on neurocognitive testing. At the time of cognitive decline, imaging studies show reduced hippocampal volume, medial temporal lobe atrophy, white matter lesions, and other non-specific changes on MRI, although these measures lack sensitivity and specificity and are thus not recommended for AD diagnosis (Knopman et al., 2021). MRI or CT imaging is mainly utilized to rule out other non-AD brain pathology that may lead to memory loss or in cases where the diagnosis of AD is not clear. PET-CT imaging of Aβ plaques has been used in research protocols (Roe et al., 2010), but is not widely utilized in diagnosis. AD features on neurocognitive testing or brain imaging are typical of patients that already have significant disease progression and are therefore limited in studying the prevention of AD. Currently, there are no recommendations for the routine use of biomarker screening tools to detect AD. However, the potential use of retinal amyloid as an early indicator of which patients to further screen may lead to earlier diagnosis. Since Aβ plaques predate cognitive decline by decades, monitoring retinal plaques and other AD pathology over time may help to establish which patients will advance to MCI or AD. Hence, the development and validation of the most effective retinal biomarkers and imaging technologies is still an active area of investigation. Our AD-RO model provides human tissue that may be used to discover novel retinal biomarkers.

Moreover, currently there are no treatments for AD that can significantly slow disease progression. Models that are amenable to drug development and that can recapitulate human pathophysiology are critical for the development of effective treatments. High-throughput drug discovery usually begins with 2D cultures of immortalized cell lines. However, 2D neuronal cultures from AD patients do not generate extracellular Aβ plaques, while 3D brain organoids do, highlighting the importance of the 3D structure for disease modeling (Kondo et al., 2013). Even though brain organoids are of obvious utility in AD research, we postulate that ROs can be a useful tool in the effort toward developing new therapies for AD. Specifically, ROs have many of the same advantages as brain organoids, as well as additional benefits, including: a fairly consistent cellular composition, which is important in quantitative studies; a transparent structure that facilitates fluorescence-based outcome measures in whole tissues; and the availability of validated technologies for outcome measure quantification in whole organoids (Vergara et al., 2017). While it is important to recognize that, to date, organoid models are not ideal for true high-throughput assays for drug discovery, because their generation is long and costly and requires manual labor, they provide an opportunity to test hits identified in simplified high-throughput screens on 3D retinal tissues prior to animal testing, thus enhancing the drug development pipeline and reducing the number of ineffective molecules that advance to animal testing and clinical trials, with their associated costs (Aasen and Vergara, 2020). Technologies for automation and standardization of organoid cultures are actively being developed by many groups and may 1 day increase the throughput of organoid assays.

Other limitations of RO (and brain organoid) models include the lack of associated structures such as vasculature, and thus the inability to model the blood–retinal barrier. Additionally, microglia, a yolk sac-derived cell type, do not develop in RO cultures. Since Aβ plaques are typically associated with microgliosis, and microglia participate in Aβ clearance, ROs could be used to evaluate drugs that may, for example, affect Aβ deposition, but they would not be a good model to evaluate drugs that may enhance its clearance.

Finally, the AD-RO model may be used to further our basic understanding of the pathology of AD in the retina and how it may impact vision. The fact that organoid models are simplified systems that lack associated structures could be exploited in testing the role of individual factors (e.g., cytokines, metabolites, or others) on AD histopathology by precisely manipulating their concentration in the media without additional confounders.

In summary, this novel AD-RO model will constitute a valuable tool in the repertoire to advance translational research on AD.

## Data availability statement

The original contributions presented in this study are included in this article/Supplementary material, further inquiries can be directed to the Corresponding author.

## Ethics statement

Ethical approval was not required for the studies on humans in accordance with the local legislation and institutional requirements because only commercially available or previously established cell lines were used.

## Author contributions

EJ: Data curation, Formal Analysis, Investigation, Methodology, Writing – original draft. AV: Formal analysis, Investigation, Methodology, Writing – original draft, Writing – review and editing. KC: Formal analysis, Investigation, Methodology, Writing – original draft, Data curation, Writing –review & editing. HL: Investigation, Methodology, Formal analysis, Writing – original draft. BL: Investigation, Methodology, Writing – review & editing. SP: Methodology, Writing – review & editing, Investigation. AH: Writing – review & editing, Methodology. NJ: Writing – review & editing, Investigation. HC: Conceptualization, Writing – review & editing. HP: Conceptualization, Writing – review & editing. MV: Conceptualization, Data curation, Formal analysis, Funding acquisition, Project administration, Resources, Writing – original draft, Writing – review & editing.

## Supplementary material

Supplementary Figure 1 Alzheimer’s disease retinal organoids produce late born cells. At 5 months of differentiation, immunofluorescence with antibodies to later born retinal cell types show: photoreceptors (RxRγ: **a,e**) and (NRL: **b,f**); rod bipolar cells (PKCa: **c,g**); and Muller glia (CRALBP: **d,h**) are all present in AD-ROs and CTR-ROs. **(i)** Quantification of RXRγ and NRL show no differences between AD-ROs and CTR-ROs (*n* = 5 per condition). Cell lines used were A18945 (CTR) and HVRDi001-A-1 (AD). Scale bars: 50 μm. Bar graphs represent mean ± SEM.
