# Supplementary figures and images for: Human iPSC-derived retinal organoids develop robust Alzheimer’s disease neuropathology
Source: Front Cell Neurosci. 2024 Jan 23;18:1340448. doi: 10.3389/fncel.2024.1340448 (PMC10844524; doi:10.3389/fncel.2024.1340448)

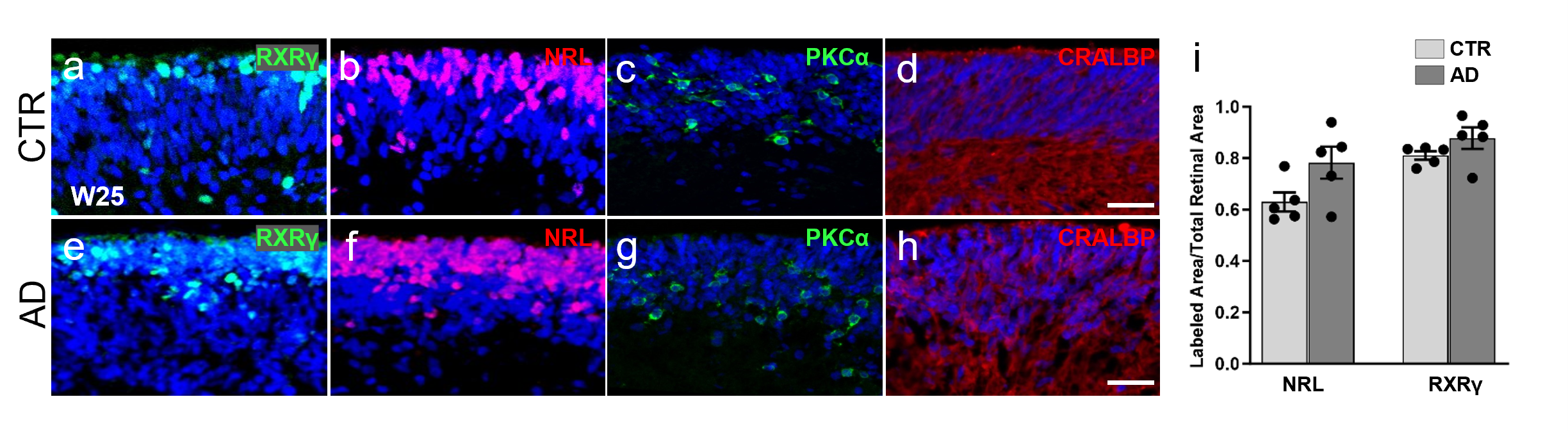

Supplement: Supplementary Figure 1 — Alzheimer’s disease retinal organoids produce late born cells. At 5 months of differentiation, immunofluorescence with antibodies to later born retinal cell types show: photoreceptors (RxRγ: a,e) and (NRL: b,f); rod bipolar cells (PKCa: c,g); and Muller glia (CRALBP: d,h) are all present in AD-ROs and CTR-ROs. (i) Quantification of RXRγ and NRL show no differences between AD-ROs and CTR-ROs (n = 5 per condition). Cell lines used were A18945 (CTR) and HVRDi001-A-1 (AD). Scale bars: 50 μm. Bar graphs represent mean ± SEM. [file Image_1.tif]
